# Supplementary material for: Air quality related equity implications of U.S. decarbonization policy
Source: Nat Commun. 2023 Sep 19;14:5543. doi: 10.1038/s41467-023-41131-x (PMC10509219; doi:10.1038/s41467-023-41131-x)
Supplement: Supplementary file 1 — Supplementary Information [file 41467_2023_41131_MOESM1_ESM.pdf]

Supplementary Information for

Air Quality Related Equity Implications of U.S. Decarbonization Policy

Paul Picciano,[1*] Minghao Qiu,[2,3*] Sebastian Eastham,[4,5] Mei Yuan,[5] John Reilly,[5] Noelle E. Selin[1,6^]

1 Institute for Data, Systems, and Society, Massachusetts Institute of Technology, Cambridge, MA 02139, United States of America
2 Doerr School of Sustainability, Stanford University, Stanford, CA, 94305, United States of America
3 Center for Innovation in Global Health, Stanford University, Stanford, CA, 94305, United States of America
4 Laboratory for Aviation and the Environment, Department of Aeronautics and Astronautics, Massachusetts Institute of Technology, Cambridge, MA 02139, United States of America
5 Joint Program on the Science and Policy of Global Change, Center for Global Change Science, Massachusetts Institute of Technology, Cambridge, MA 02139, United States of America
6 Department of Earth, Atmospheric and Planetary Sciences, Massachusetts Institute of Technology, Cambridge, MA 02139, United States of America

* These authors contributed equally to this work.
^ Corresponding author: selin@mit.edu

Figure S1. Change in $PM_{2.5}$ concentrations under Cap 50% (2030) relative to Baseline (2030), due to emission changes from the six sectors. National population-weighted averages of the $PM_{2.5}$ changes from each sector's emissions are listed under each respective title. Please note the different color scales for each sector. The base map of U.S. states is plotted using the R package tigris (https://CRAN.R-project.org/package=tigris), with original shape files from the U.S. Census Bureau (https://www.census.gov/geographies/mapping-files/time-series/geo/tiger-line-file.html, year 2019, resolution 20m).

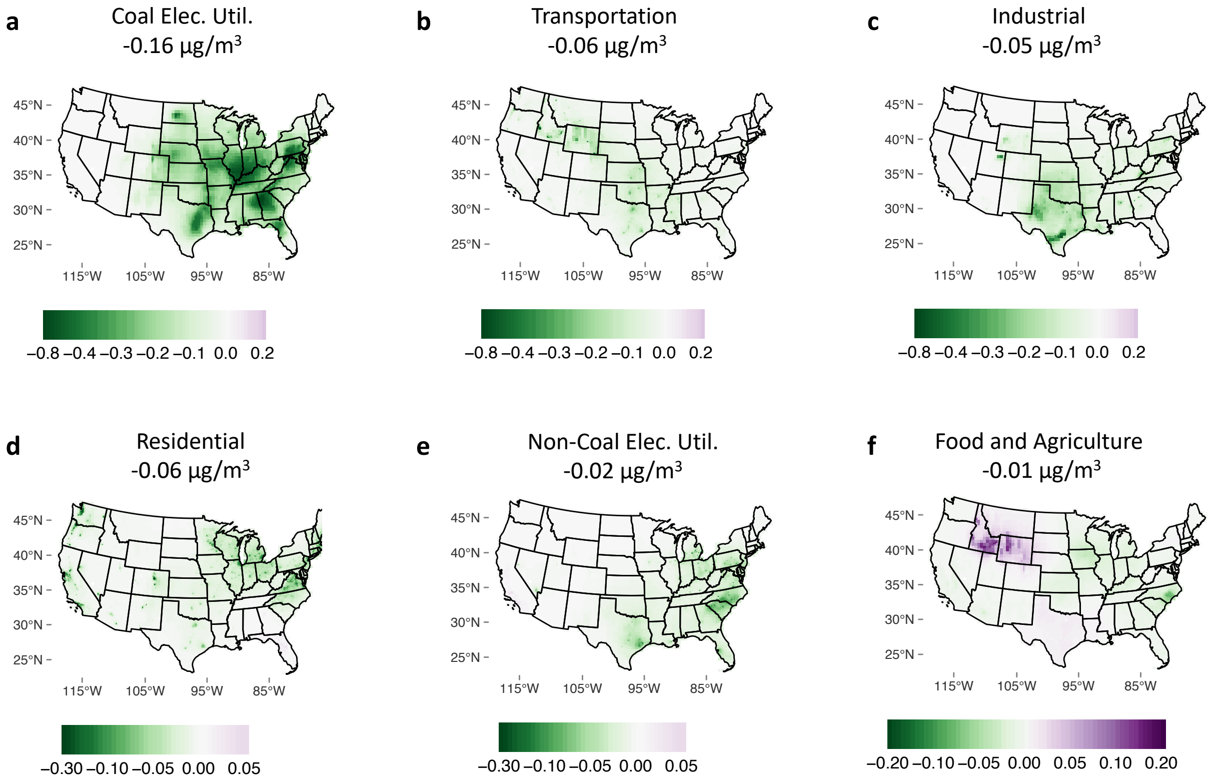

Figure S2. State-level percentage point changes in disparity under Cap 50% (2030) relative to Baseline (2030) (i.e. absolute change in relative percentage disparity) for each population group. Changes in national relative disparity for each population group are provided under each population name. Racial/ethnic categories are derived from the American Community Survey. The base map of U.S. states is plotted using the R package tigris (https://CRAN.R-project.org/package=tigris), with original shape files from the U.S. Census Bureau (https://www.census.gov/geographies/mapping-files/time-series/geo/tiger-line-file.html, year 2019, resolution 20m).

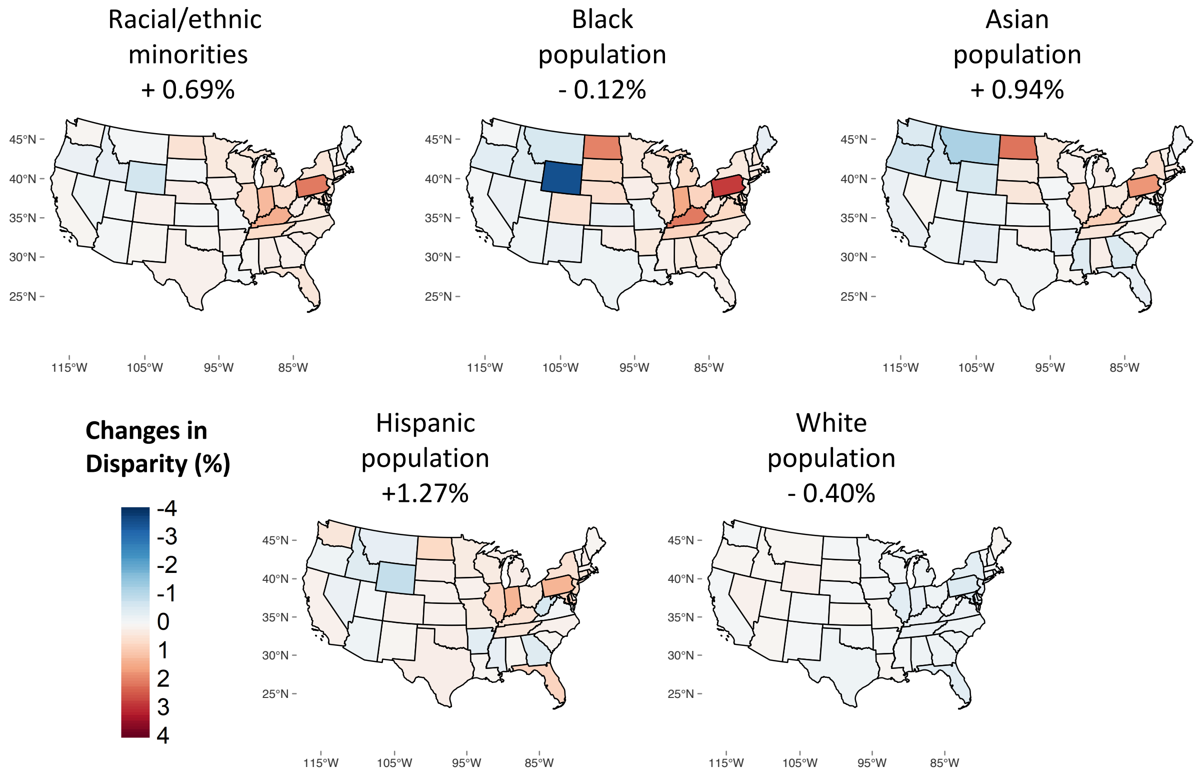

Figure S3. Impacts of carbon policy on exposure disparities for each ethnic/racial group in the 20 major urbanized areas in the US. The dots show the changes in within-city disparity due to the simulated carbon policy scenario (cap 50% (2030)) relative to the baseline scenarios for each of the 20 urbanized areas. The triangle shows the impacts on the average disparities pooling across the 20 urbanized areas (i.e. the estimates shown in Figure 3, but only for these 20 urbanized areas).

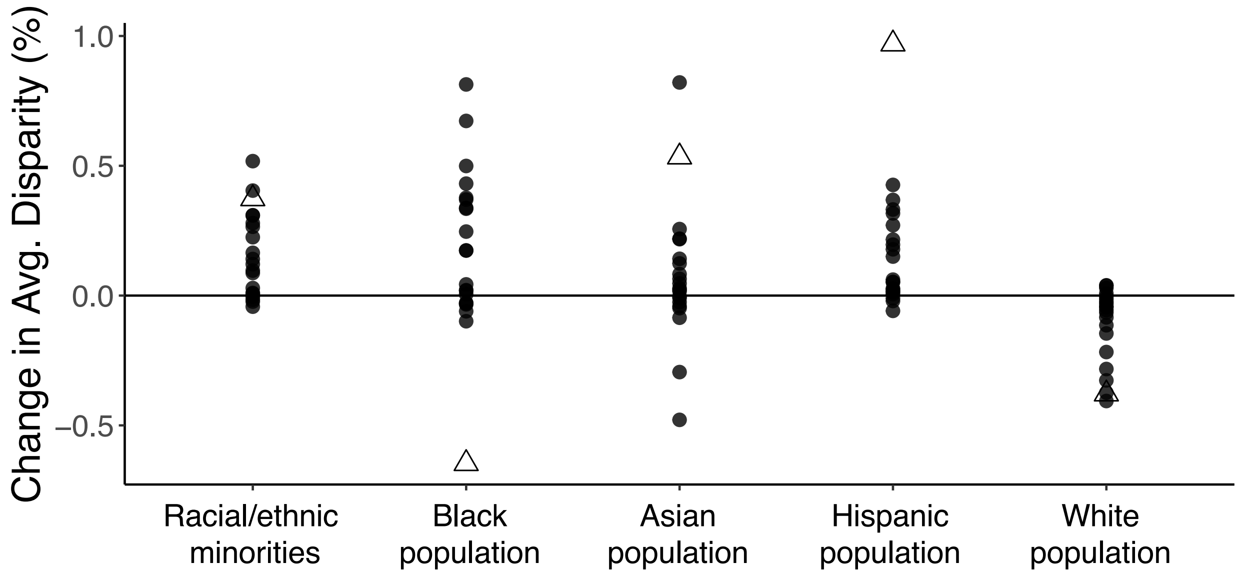

Figure S4. Sectoral contributions to the $PM_{2.5}$ exposure of racial/ethnic minority group under alternative CO_2 emission reduction distributions. These optimizations scenarios minimize $PM_{2.5}$ mortality of racial/ethnic minorities while keeping overall CO_2 reductions constant for different region/sector combinations.

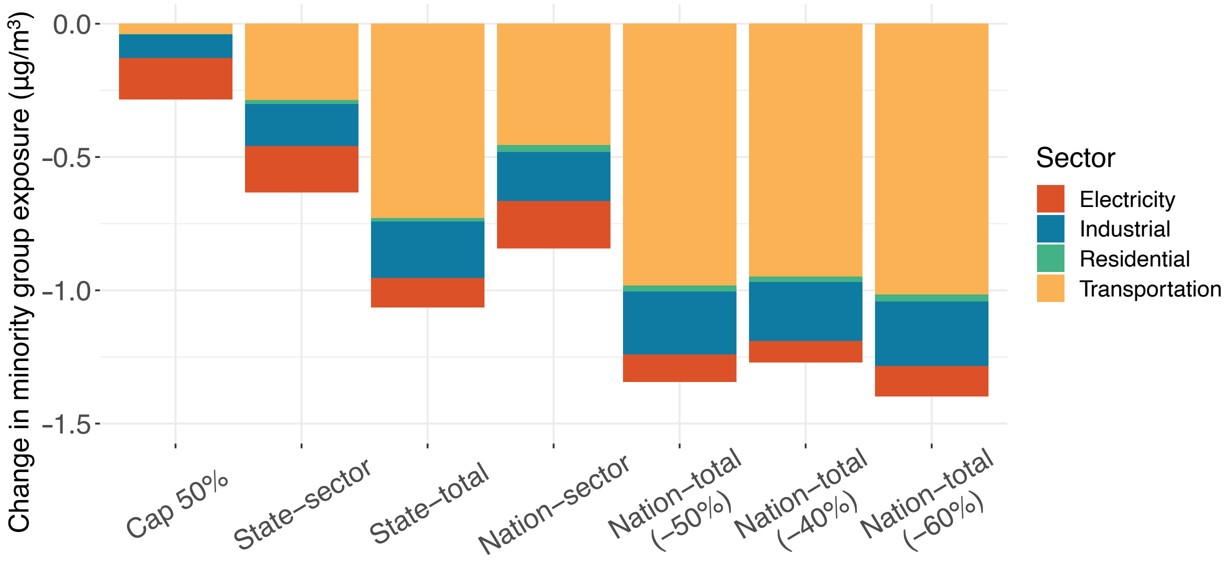

Table S1: Impacts of carbon policy on exposure disparities for each racial/ethnic group within the 20 major urbanized areas in the US. The figure shows the existing pollution disparities within each city (under the 2030 baseline scenario), and the changes in disparity due to the simulated carbon policy scenario (cap 50% (2030)).

	Asian population		*Black* population		*Hispanic* population		*Racial/ethnic minorities*		*White* population	
	baseline disparity	*Δ disparity*	*baseline disparity*	*Δ disparity*	*baseline disparity*	*Δ disparity*	*baseline disparity*	*Δ disparity*	*baseline disparity*	*Δ disparity*
New York--Newark, NY--NJ--CT	*9.5%*	*0.26%*	*10.0%*	*0.33%*	*12.0%*	*0.32%*	*10.7%*	*0.31%*	*-11.3%*	*-0.33%*
Los Angeles--Long Beach--Anaheim, CA	*-5.2%*	*-0.03%*	*9.0%*	*-0.03%*	*5.8%*	*0.03%*	*3.4%*	*0.01%*	*-8.3%*	*-0.02%*
Chicago, IL--IN	*2.2%*	*0.08%*	*12.4%*	*0.02%*	*7.7%*	*0.18%*	*8.5%*	*0.10%*	*-7.4%*	*-0.08%*
Miami, FL	*-8.3%*	*0.02%*	*1.8%*	*0.01%*	*14.4%*	*-0.01%*	*9.2%*	*0.00%*	*-17.0%*	*0.01%*
Houston, TX	*5.5%*	*-0.09%*	*1.2%*	*-0.06%*	*1.9%*	*0.01%*	*2.0%*	*-0.02%*	*-3.5%*	*0.04%*
Dallas--Fort Worth--Arlington, TX	*5.9%*	*0.22%*	*-1.8%*	*-0.10%*	*0.0%*	*0.01%*	*0.1%*	*0.00%*	*-0.1%*	*0.00%*
Philadelphia, PA--NJ--DE--MD	*5.3%*	*0.22%*	*15.6%*	*0.67%*	*11.5%*	*0.43%*	*12.4%*	*0.52%*	*-6.8%*	*-0.28%*
Washington, DC--VA--MD	*-4.1%*	*-0.48%*	*6.1%*	*0.81%*	*2.6%*	*0.05%*	*2.8%*	*0.31%*	*-3.4%*	*-0.37%*
Atlanta, GA	*3.0%*	*0.82%*	*3.6%*	*0.38%*	*2.6%*	*0.37%*	*3.2%*	*0.40%*	*-3.2%*	*-0.41%*
Boston, MA--NH--RI	*16.2%*	*0.14%*	*27.0%*	*0.17%*	*21.3%*	*0.20%*	*20.3%*	*0.16%*	*-6.8%*	*-0.06%*
Phoenix--Mesa, AZ	*0.2%*	*0.02%*	*4.3%*	*-0.03%*	*6.2%*	*-0.06%*	*5.1%*	*-0.04%*	*-3.5%*	*0.03%*
Detroit, MI	*-1.3%*	*-0.05%*	*10.3%*	*0.34%*	*4.8%*	*0.33%*	*7.9%*	*0.28%*	*-4.1%*	*-0.15%*
Seattle, WA	*13.7%*	*-0.01%*	*6.9%*	*0.43%*	*0.7%*	*0.02%*	*5.8%*	*0.09%*	*-2.8%*	*-0.04%*

San Francisco--Oakland, CA	*-1.4%*	*0.00%*	*15.3%*	*0.04%*	*6.9%*	*0.02%*	*3.9%*	*0.01%*	*-6.3%*	*-0.01%*
San Diego, CA	*3.5%*	*-0.04%*	*5.5%*	*0.02%*	*-1.6%*	*0.06%*	*0.4%*	*0.03%*	*-0.4%*	*-0.03%*
Minneapolis--St. Paul, MN--WI	*6.8%*	*0.12%*	*11.5%*	*0.17%*	*8.5%*	*0.15%*	*8.6%*	*0.14%*	*-2.7%*	*-0.04%*
Tampa--St. Petersburg, FL	*1.3%*	*0.06%*	*5.1%*	*0.25%*	*6.3%*	*0.27%*	*5.0%*	*0.22%*	*-2.5%*	*-0.11%*
Denver--Aurora, CO	*-0.9%*	*0.03%*	*16.7%*	*0.50%*	*6.7%*	*0.05%*	*7.0%*	*0.12%*	*-3.8%*	*-0.07%*
Riverside--San Bernardino, CA	*-3.3%*	*0.05%*	*-0.2%*	*-0.03%*	*6.4%*	*-0.02%*	*4.3%*	*-0.02%*	*-10.6%*	*0.04%*
Baltimore, MD	*-6.3%*	*-0.29%*	*7.3%*	*0.37%*	*2.4%*	*0.22%*	*4.9%*	*0.27%*	*-4.1%*	*-0.22%*

Table S2. Impacts of carbon policy on change in $PM_{2.5}$ exposure and change in average disparity (percentage points) of racial/ethnic minorities, relative to Baseline 2030 scenario, for each policy scenario.

Scenario number	*Policy case (CO_2 targets)*	Scenario	Change in minority group exposure ($\mu g\ m^{-3}$)	Change in average disparity
3	*-50% relative to 2005*	*Cap 50% (2030)*	*-0.36*	*+ 0.7%*
5	*-50% relative to 2005*	*Nation-sector*	*-0.84*	*-1.9%*
6	*-50% relative to 2005*	*State-total*	*-1.06*	*-0.3%*
7	*-50% relative to 2005*	*State-sector*	*-0.63*	*-0.5%*
8	*-50% relative to 2005*	*Nation-total*	*-1.34*	*-2.0%*
9	*-40% relative to 2005*	*Nation-total (-40%)*	*-1.20*	*-2.7%*
10	*-60% relative to 2005*	*Nation-total (-60%)*	*-1.43*	*-1.5%*
