# Supplementary material for: Air quality related equity implications of U.S. decarbonization policy
Source: Nat Commun. 2023 Sep 19;14:5543. doi: 10.1038/s41467-023-41131-x (PMC10509219; doi:10.1038/s41467-023-41131-x)
Supplement: Supplementary file 3 — Reporting Summary [file 41467_2023_41131_MOESM3_ESM.pdf]

Corresponding author(s): Selin

Last updated by author(s): 11 August 2023

Reporting Summary

Nature Portfolio wishes to improve the reproducibility of the work that we publish. This form provides structure for consistency and transparency in reporting. For further information on Nature Portfolio policies, see our Editorial Policies and the Editorial Policy Checklist.

Statistics

For all statistical analyses, confirm that the following items are present in the figure legend, table legend, main text, or Methods section.

n/a	Confirmed	
☐	☒	The exact sample size (n) for each experimental group/condition, given as a discrete number and unit of measurement
☒	☐	A statement on whether measurements were taken from distinct samples or whether the same sample was measured repeatedly
☒	☐	The statistical test(s) used AND whether they are one- or two-sided<br>*Only common tests should be described solely by name; describe more complex techniques in the Methods section.*
☒	☐	A description of all covariates tested
☒	☐	A description of any assumptions or corrections, such as tests of normality and adjustment for multiple comparisons
☐	☒	A full description of the statistical parameters including central tendency (e.g. means) or other basic estimates (e.g. regression coefficient) AND variation (e.g. standard deviation) or associated estimates of uncertainty (e.g. confidence intervals)
☒	☐	For null hypothesis testing, the test statistic (e.g. F, t, r) with confidence intervals, effect sizes, degrees of freedom and P value noted<br>*Give P values as exact values whenever suitable.*
☒	☐	For Bayesian analysis, information on the choice of priors and Markov chain Monte Carlo settings
☒	☐	For hierarchical and complex designs, identification of the appropriate level for tests and full reporting of outcomes
☒	☐	Estimates of effect sizes (e.g. Cohen's d, Pearson's r), indicating how they were calculated

Our web collection on statistics for biologists contains articles on many of the points above.

Software and code

Policy information about availability of computer code

Data collection	MIT US Regional Energy Policy (USREP) model, published by MIT Joint Program on the Science and Policy of Global Change (sources cited in the paper);<br>InMAP Source Receptor Matrix, published by Goodkind et al (sources cited in the paper);<br>National Emission Inventory (NEI) 2017, published by U.S. Environmental Protection Agency;<br>Block-group level population data by race/ethnicity, derived from the 5-Year 2012 American Community Survey (ACS);<br>Population projection data in 2030, published by University of Virginia (sources cited in the paper).
Data analysis	Models used in this project are available via links in published references. Original code (e.g. for analysis/plotting) is described fully in manuscript, and is publicly available from https://doi.org/10.5281/zenodo.8226507. Data analysis is primarily performed using programming language R (version 4.3.0)

For manuscripts utilizing custom algorithms or software that are central to the research but not yet described in published literature, software must be made available to editors and reviewers. We strongly encourage code deposition in a community repository (e.g. GitHub). See the Nature Portfolio guidelines for submitting code & software for further information.

Data

Policy information about availability of data

All manuscripts must include a data availability statement. This statement should provide the following information, where applicable:

- Accession codes, unique identifiers, or web links for publicly available datasets
- A description of any restrictions on data availability
- For clinical datasets or third party data, please ensure that the statement adheres to our policy

All data that support the findings of the paper is presented in the paper, supplementary information, and public data repo. Main results, input data and code scripts that can replicate the main figures of the paper can be accessed from: https://zenodo.org/record/8226507

Human research participants

Policy information about studies involving human research participants and Sex and Gender in Research.

Reporting on sex and gender	n/a
Population characteristics	n/a
Recruitment	n/a
Ethics oversight	n/a

Note that full information on the approval of the study protocol must also be provided in the manuscript.

Field-specific reporting

Please select the one below that is the best fit for your research. If you are not sure, read the appropriate sections before making your selection.

☐ Life sciences ☐ Behavioural & social sciences ☒ Ecological, evolutionary & environmental sciences

For a reference copy of the document with all sections, see nature.com/documents/nr-reporting-summary-flat.pdf

Ecological, evolutionary & environmental sciences study design

All studies must disclose on these points even when the disclosure is negative.

Study description	Modeling experiments were used, including optimization.
Research sample	Data used include results from simulations of emissions, air quality, combined with population data over continental U.S.
Sampling strategy	No sampling within the study focus was conducted: data reflects model results applied to the entire continental U.S.
Data collection	No raw data is collected in the study.
Timing and spatial scale	The model covers the continental U.S. and calculates the annual average conditions in year 2017 and 2030.
Data exclusions	No data excluded.
Reproducibility	Models employed are previously published and available openly; analysis code is made available publicly.
Randomization	This study did not use experimental procedures that involve randomization and controls.
Blinding	This study did not use experimental procedures that involve evaluating a test vs a control, and thus blinding is not applicable.

Did the study involve field work? ☐ Yes ☒ No

Reporting for specific materials, systems and methods

We require information from authors about some types of materials, experimental systems and methods used in many studies. Here, indicate whether each material, system or method listed is relevant to your study. If you are not sure if a list item applies to your research, read the appropriate section before selecting a response.

Materials & experimental systems

n/a	Involved in the study
☒	☐ Antibodies
☒	☐ Eukaryotic cell lines
☒	☐ Palaeontology and archaeology
☒	☐ Animals and other organisms
☒	☐ Clinical data
☒	☐ Dual use research of concern

Methods

n/a	Involved in the study
☒	☐ ChIP-seq
☒	☐ Flow cytometry
☒	☐ MRI-based neuroimaging
